# Molecular diagnosis of strongyloidiasis in a population of an endemic area through nested-PCR 

**Published:** 2018

**Authors:** Meysam Sharifdini, Amir Keyhani, Mohammad Reza Eshraghian, Eshrat Beigom Kia

**Affiliations:** 1 *Department of Medical Parasitology and Mycology, School of Medicine, Guilan University of Medical Sciences, Rasht, Iran*; 2 *Department of Medical Parasitology and Mycology, School of Public Health, Tehran University of Medical Sciences, Tehran, Iran*; 3 *Department of Biostatistics and Epidemiology, School of Public Health, Tehran University of Medical Sciences, Tehran, Iran*

**Keywords:** elderly, molecular diagnosis, nested-PCR, *Strongyloides stercoralis*

## Abstract

**Aim:**

This study is aimed to diagnose and analyze strongyloidiasis in a population of an endemic area of Iran using nested-PCR, coupled with parasitological methods.

**Background:**

Screening of strongyloidiasis infected people using reliable diagnostic techniques are essential to decrease the mortality and morbidity associated with this infection. Molecular methods have been proved to be highly sensitive and specific for detection of *Strongyloides stercoralis* in stool samples.

**Methods:**

A total of 155 fresh single stool samples were randomly collected from residents of north and northwest of Khouzestan Province, Iran. All samples were examined by parasitological methods including formalin-ether concentration and nutrient agar plate culture, and molecular method of nested-PCR. Infections with *S. stercoralis* were analyzed according to demographic criteria.

**Results:**

Based on the results of nested-PCR method 15 cases (9.7%) were strongyloidiasis positive. Nested-PCR was more sensitive than parasitological techniques on single stool sampling. Elderly was the most important population index for higher infectivity with *S. stercoralis*.

**Conclusion:**

In endemic areas of *S. stercoralis*, old age should be considered as one of the most important risk factors of infection, especially among the immunosuppressed individuals.

## Introduction

 Strongyloidiasis is an intestinal infection caused by nematode *Strongyloides stercoralis*. It is one of the most important neglected soil-transmitted helminth infections that is prevalent in tropical and temperate regions with poor sanitation standards (1).

Transmission of *S. stercoralis* to human is mainly by penetration of infective third-stage larvae through the skin while contacts with the soil (1). Strongyloidiasis has variable manifestations from asymptomatic infections to mild gastrointestinal, cutaneous, and pulmonary symptoms with or without fever. In addition, chronic infection may lead to hyperinfection and disseminated infection in immunosuppressed individuals, transplant recipients and those receiving corticosteroid treatment that may be fatal if not treated adequately (2, 3). With the increasing number of immunocompromised individuals around the world, severe complicated strongyloidiasis could pose a major health problem to these patients (4). Therefore, screening of infected people using reliable diagnostic techniques is essential for the detection of the parasite in people at risk in order to decrease the mortality and morbidity associated with this infection. 

Various parasitological techniques have been used for the diagnosis of *S. stercoralis* in fecal samples, such as formalin–ether concentration, nutrient agar plate culture, Harada-Mori culture and Baermann method (5). Diagnosis of strongyloidiasis using parasitological methods is difficult because in most cases parasitic load is low and the larval output is minimal (4). Several studies have shown that the nutrient agar plate culture is more sensitive than other parasitological techniques towards the detection of larvae in stool samples (6, 7). However, this technique is laborious, time consuming (requiring 2–3 days), and also requires fresh stool samples and experienced laboratory technicians (5, 7). Sensitivity of parasitological techniques can be increased with multiple stool sampling over consecutive days (8). 

Molecular methods have proved to be highly sensitive and specific for detection of parasitic agents in fecal samples (9-11). In a study carried out by Sharifdini *et al.* on molecular diagnosis of strongyloidiasis, the sensitivity and specificity of nested-PCR have been reported 100% and 91.6%, respectively (12). Moreover, application of molecular methods is also found to decrease the number of serial stool samples necessary to give a diagnosis of strongyloidiasis with the maximum sensitivity (13).

Khouzestan Province in southwest of Iran has previously been perceived as the endemic area for strongyloidiasis and hookworm infections (14). However, in recent decades human hookworm infections have sharply declined, while strongyloidiasis due to capability of autoinfection still remains of public health concern and even fatal cases has been reported from residents in this province (15). This study is aimed to diagnose strongyloidiasis infection in a population of north and northeast of Khouzestan Province, using nested-PCR, coupled with parasitological methods. 

## Methods

**Study area and sampling**

 Khuzestan Province in southwest of Iran is situated between 48°E and 49.5°E longitudes and between 31°N and 32°N latitudes, bordering Iraq and Persian Gulf (Fig. 1). The study area, the northern part of this Province, includes three cities namely Andimeshk, Dezful and Shoush that lies close to the foothills of the Zagros Mountains. The annual amounts of rainfall in this area is between 995 and 1100 mm. This area has a hot and dry climate with extremely hot summers and warm winters (16). 

From April 2012 to March 2013, a total of 155 fresh single stool samples were randomly collected from residents of this study area. Demographic data of participants were recorded in a questionnaire. All the samples were examined using parasitological methods and nested-PCR

**Parasitological methods**

All stool samples were examined using formalin-ether concentration as well as nutrient agar plate culture methods. For nutrient agar plate culture, as described previously (7, 17), 3–4 g of fresh stool sample was placed on the center of the plate. After incubation at 28–30°C for 48-72 h, the plates were checked using stereomicroscope. If any larva, adults or their tracks were seen, surface of the plate was washed by lukewarm phosphate buffer saline solution (7). Then, morphological characteristics of parasites were observed to differentiate *S. stercoralis* from other possible nematodes including hookworms, *Trichostrongylus* spp. and *Rhabditis* spp. (18).

**DNA extraction**

For extraction of genomic DNA from stool samples, a part of each sample was preserved in 70% ethanol alcohol at room temperature. DNA extraction was performed using an in-house method as described by Sharifdini *et al.* (12). The samples were washed with sterile distilled water to remove ethanol. Then, about one gram of each stool diluted in 10 mL PBS followed by freezing and thawing for five cycles. Next, approximately 500 μL PBS-diluted stool was incubated overnight in 500 μL GTES buffer (100 mM glycine, 0.05% SDS, 100 mM Tris/Cl, and 1 mM EDTA) at 37°C. Afterwards, the samples were subjected to three freeze–thaw cycles, 200 mg of glass beads were then added to the samples and shaken vigorously. The supernatant was incubated in nematode lysis buffer (100 mM EDTA, 100 mM NaCl, 100 mM Tris pH 7.5, 0.05% SDS, proteinase K 100 μg/mL) for 12 hours at 37°C, the samples were then extracted with one volume of phenol–chloroform–isoamyl alcohol (25:24:1) for two times, and the DNA extract was precipitated by an equal volume of isopropanol and 1 mL of absolute ethanol, respectively. The pellet was washed with 300 μL of 70% ethanol, suspended in 100 μL of Tris/EDTA buffer. Extracted DNA samples were kept at −20°C until use.

**Nested-PCR**

Nested-PCR protocol for amplification of a partial mitochondrial cytochrome c oxidase subunit 1 (cox1) gene of *S. stercoralis* was carried out based on the method described by Sharifdini *et al.* (12). For the first amplification round, external primers namely COXF (5′- TGGTTTGGGTACTAGTTG -3′) and COXR (5′- GATGAGCTCAAACTACACA -3′) were used, which produced a 509-bp target, and for the second amplification round the internal CNF (5′-TTCTAGTGTTGATTTGGC T-3′) and CNR (5′-TTACCACCAAAACTAGGATC-3′) were used to amplify a 261-bp internal fragment. The PCR amplification was carried out in a final reaction volume of 20 µl containing 10 μl of PCR mix included of 1.25 U Taq DNA polymerase, 200 μM of dNTPs and 1.5 mM MgCl2 (2x Master Mix RED Ampliqon, Denmark), 10 pmol of each primer and 4 μL of DNA sample for the first PCR round, and 1 μL of the first PCR product (diluted 1/40) as a template for the second round. Distilled water (instead of DNA template) as the negative control was included in each PCR run. The cycling conditions for first round of PCR reaction were an initial denaturation step at 95°C for 6 minutes followed by 35 cycles of 95°C for 45 seconds (denaturation), 55°C for 60 seconds (annealing), and at 72°C for 60 seconds (extension) with a final extension of 72°C for 6 minutes. PCR conditions for the second round comprised of 95 °C for 2 min, followed by 25 cycles of 94 °C for 15 seconds, 60 °C for 30 seconds, and 70 °C for 30 seconds, plus a final extension at 72 °C for 6 minutes. Subsequently, 5 μL of each nested-PCR product was electrophoresed on a 1.5% agarose gel and visualized using a UV transilluminator after staining with 0.5 μg/mL ethidium bromides.

**Data analysis**

Data processing and analysis were carried out using SPSS software (Statistical Package for Social Sciences), version 18 by Chi-square and Fisher exact tests. A P-value of less than 0.05 was considered as statistically significant difference. 

## Results

Out of 155 individuals examined, 87 were male and 68 were female. Age of participants ranged from 2 to 83 years old with a mean age of 29.0 ±17.7 years. With respect to the results of infectivity with *S. stercoralis *(Table 1), 1 (0.64 %) and 5 (3.2%) people were found infected using formalin-ether concentration and agar plate culture, respectively. The case found positive by formalin-ether concentration, was also detected by agar plate culture, as well as nested-PCR. An 83 years old patient with rheumatoid arthritis, who was under a long corticosteroid therapy was found to have hyperinfection strongyloidiasis syndrome which exhibited clinical manifestations of epigastric pain, abdominal discomfort, intermittent diarrhea, constipation and nausea. 

Nested-PCR showed 15 (9.7%) samples positive for *S. stercoralis* (Table 1). This method confirmed all cases found positive by formalin-ether concentration and agar plate culture methods. In addition, 9 more cases which were negative by the parasitological methods were found positive by nested-PCR. Therefore, results of nested-PCR (15 cases) were used as the basis of analysis of infectivity with *S. stercoralis*. 

Table 2 illustrates the distribution of strongyloidiasis cases among the study population from Khouzestan Province, based on nested-PCR results, according to demographic criteria including age groups, gender, educational status and occupation of participants. Statistical analysis revealed that there was a significant difference between age groups and strongyloidiasis (*P*=0.04), so that the age group of more than 60 years was more infected than other age groups. However, no significant difference was found between the infection and any other criteria including gender, educational status and occupation of individuals.

**Table 1:** Demographic characterization and diagnostic tests results for strongyloidiasis infected cases in a study population (n = 155) of Khouzestan Province, south-west Iran

Case order	Sex	Age (year)	Educational status	Occupation	Location	Diagnostic tests		
						Formalin-ether concentration	Agar plate culture	Nested-PCR
1	M	31	Diploma	Others	City	-	-	+
2	M	73	Illiterate	Others	City	-	-	+
3	M	26	Diploma	Farmer	Village	-	-	+
4	M	29	Primary school	Shepherd	Village	-	-	+
5	F	15	Secondary school	Student	Village	-	-	+
6	M	49	Diploma	Others	City	-	-	+
7	M	17	Secondary school	Student	City	-	-	+
8	F	44	Primary school	House wife	Village	-	-	+
9	M	77	Illiterate	Others	Village	-	-	+
10	F	73	Primary school	House wife	Village	-	-	+
11^*^	F	83	Illiterate	House wife	Village	+	+	+
12	F	5	Illiterate	Children under school age	Village	-	+	+
13	M	23	Diploma	Farmer	Village	-	+	+
14	M	10	Primary school	Student	Village	-	+	+
15	M	40	Primary school	Farmer	Village	-	+	+

M = male, F = female,

* this case had hyperinfection syndrome

**Table 2:** Rate of strongyloidiasis using nested-PCR in a population of Khouzestan Province, south-west Iran according to demographic criteria

Variables	Infection (n=15) (9.7%)	No. examined (155)	P-value
Age group (year)			P = 0.040
<15	3 (6.4)	47	
15-30	4 (10.3)	39	
31-45	3 (7.3)	41	
46-60	1 (5.3)	19	
>60	4 (44.4)	9	
Gender			P = 0.427
Male	10 (11.5)	87	
Female	5 (7.4)	68	
Educational status			P = 0.378
Illiterate	4 (18.2)	22	
Primary school	5 (10.4)	48	
Secondary school	2 (5.9)	34	
Diploma	4 (11.8)	34	
Collage and above	0 (0)	17	
Occupation			P = 0.384
Employer	0 (0)	12	
Farmer	3 (25)	12	
Shepherd	1 (14.3)	7	
Worker	0 (0)	10	
House wife	3 (8.8)	34	
Children under school age	1 (7.7)	13	
Student	3 (7)	43	
Others	4 (16.6)	24	
Location			P = 0.5
City	4(8.5)	47	
Village	11(10.2)	108	

## Discussion

Strongyloidiasis is important in immunocompromised patients by producing hyperinfection and disseminated syndromes and may be fatal if not treated adequately (4, 5). *S. stercoralis* is prevalent in tropical and subtropical areas of the world, infecting probably 100 million people in 70 countries (3). It is endemic in northern and southern coastal provinces of Iran that have suitable moist environment for establishment of the lifecycle of *S. stercoralis* (15). In the last decades, due to improvement in hygienic standards, increasing of health and medical graduates, proper disposal of human fecal wastes and public awareness, prevalence of most soil-transmitted helminthes have sharply declined in the country. However, *S. stercoralis* is still prevalent in the endemic areas, due to its ability of autoinfection within its life cycle in the host. 

Sensitivity and specificity of various diagnostic methods for detection of *S. stercoralis* infection are different, therefore true prevalence of the methods is frequently underestimated (19). Different studies utilizing PCR-based techniques showed variable results for detection of *S. stercoralis* DNA in fecal samples (9, 20-23). In an evaluation, nested-PCR was found to be 1.37 times better in sensitivity and better in reliability compared to parasitological methods in detection of *S. stercoralis *in stool samples (12). In the current study, based on nested-PCR results 9.7% of a population in north and northwest of Khouzestan Province were infected with *S. stercoralis*. In other studies in Iran, the infection rate of this parasite is different according to target population and diagnostic methods utilized. Several parasitological surveys of *S. stercoralis* have been recently conducted in endemic areas of Iran. Kia *et al.* 2007 showed that 4.9% of rural inhabitants of Mazandaran Province were infected with this parasite (7). In a different study conducted in Guilan Province (24), 42% of patients with eosinophilia were infected with *S. stercoralis*. Prevalence of *S. stercoralis* was reported 17.3% in residents of a mentally retarded institution of southern Iran using formalin–ether concentration (25). This parasite was detected in 2.1% in rehabilitation centers in Mazandaran Province (26). In institutionalized mentally disabled individuals of Guilan Province, 1.2% of the residents were found infected with this nematode (27). Prevalence of *S. stercoralis* has been reported 0.6% in tribal parts of Khuzestan Province (28). Choosing a suitable diagnostic method for screening of strongyloidiasis cases is essential for estimating its actual prevalence rate which would help in its control and prevention programs. In this study, using nested-PCR, higher rate of *S. stercoralis* infection was found compared to other studies in the endemic areas of the country where parasitological methods were used (7, 28). Actually, molecular methods assist in better understanding of the *S. stercoralis* prevalence in a community due to its higher sensitivity and specificity. 

Our study has revealed that *S. stercoralis* infection was more prevalent in people over 60 years of age. It was in agreement with some previous studies found that prevalence of strongyloidiasis increases with age (15, 29). This relation may be associated with its unique ability of autoinfection in infected individuals for several decades or even their entire life. Consequently, *S. stercoralis* shows an age cumulative distribution, and is more prevalent in people of old age. Elderly, is not only a risk factor for higher infectivity with *S. stercoralis*, but also increases susceptibility to complicated infections occurrence as a result of deteriorations in organ systems and emergence of various immunosuppressive conditions, as with the case of hyperinfection in this study which happened in an 83 years old female with rheumatoid arthritis, and prolonged corticosteroid therapy. 

The results of current study illustrate that males were more infected with *S. stercoralis* than females (11.5% versus 7.4%); however, this difference was not statistically significant. This could be due to small sample size in the current study. Several researchers reported that rate of infection in male gender was higher than female (15, 28, 29), due to higher exposure of males with source of infection as a result of outdoors activities, working in the fields and gardening.

Among different demographic criteria evaluated in association with strongyloidiasis in this study, age variation showed significant impact and oldness had tendency to higher rate of infection and even overwhelming infection. Therefore, application of sensitive diagnostic methods like nested-PCR among the patients at risk with a keen attention to the people of old age in endemic areas will be of paramount importance towards the prevention of infections and their fatal consequences.
